# The Challenges and Strategies for Cleaning the Routinely Collected Data for Total Knee Replacements (TKR) and Total Hip Replacements (THR) in the Kingdom of Fife

**DOI:** 10.23889/ijpds.v9i5.2778

**Published:** 2024-09-18

**Authors:** Luciana Pedro, Amaya Azcoaga-Lorenzo, Colin McCowan

**Affiliations:** 1 School of Medicine, University of St Andrews; 2 Hospital Rey Juan Carlos; 3 Instituto de Investigación Sanitaria Fundación Jimenez Diaz

Pregnancy-related research using routinely collected data is increasing. Electronic Health Records (EHR) are a robust resource because they can provide information on a large population of pregnant women covering prolonged periods at a reasonable cost. Moreover, information is collected prospectively and is thus free from recall bias. However, these data sources also present challenges. Information is gathered for clinical purposes and often recorded in different datasets (prescriptions, hospital admission, demography, birth records, etc.). Therefore, a significant data management effort must be made to convert all the information into structured and meaningful datasets in which identifying the timing of conception, outcomes of interest, multiple pregnancies, and interpregnancy periods can easily be done.

In this study, we linked Scottish Maternity Records between 2007 and 2021 (1,575,153 pregnancies, 493,629 women) to demographics data, datasets with International Classification of Diseases (ICD) diagnostic codes for all inpatient stays and day cases in hospitals, and Prescribing Information System (PIS) for data on all medications from primary care. After combining these datasets using R codes, we developed a simple tree-based algorithm that visually represents each woman at each pregnancy and the related health conditions. Presenting data in this way helps identify relevant clinical questions and the temporal relation of different pregnancies. As an example, we identified the progression of multimorbidity through pregnancies and characterised how multimorbidity changes from one pregnancy to another, considering the woman's health conditions and social class.

